# The Prognostic Value of Amplitude-Integrated EEG in Full-Term Neonates with Seizures

**DOI:** 10.1371/journal.pone.0078960

**Published:** 2013-11-13

**Authors:** Dandan Zhang, Haiyan Ding, Lili Liu, Xinlin Hou, Guoyu Sun, Lei Li, Yunzhe Liu, Congle Zhou, Ruolei Gu, Yuejia Luo

**Affiliations:** 1 Institute of Affective and Social Neuroscience, Shenzhen University, Shenzhen, China; 2 Department of Biomedical Engineering, Tsinghua University, Beijing, China; 3 Department of Pediatrics, Peking University First Hospital, Beijing, China; 4 Key Laboratory of Cryogenics, Technical Institute of Physics and Chemistry, Chinese Academy of Science, Beijing, China; 5 Key Laboratory of Behavioral Science, Institute of Psychology, Chinese Academy of Sciences, Beijing, China; University of Alabama at Birmingham, United States of America

## Abstract

Neonatal seizures pose a high risk for adverse outcome in survived infants. While the prognostic value of amplitude-integrated electroencephalogram (aEEG) is well established in neonates with encephalopathy and asphyxia, neonatal seizure studies focusing on the direct correlation between early aEEG measurement and subsequent neurologic outcome are scarce. In this study, the prognostic value of aEEG features was systematically analyzed in 143 full-term neonates to identify prognostic indicators of neurodevelopmental outcome. Neonatal aEEG features of background pattern, cyclicity, and seizure activity, as well as the etiology of neonatal seizures, were significantly associated with neurodevelopmental outcome at one year of age. aEEG background pattern was highly associated with neurologic outcomes (χ^2^ = 116.9), followed by aEEG cyclicity (χ^2^ = 87.2) and seizure etiology (χ^2^ = 79.3). Multiple linear regression showed that the four predictors explained 71.2% of the variation in neurological outcome, with standardized β coefficients of 0.44, 0.24, 0.22, and 0.14 for the predictors of aEEG background pattern, cyclicity, etiology, and aEEG seizure activity, respectively. This clinically applicable scoring system based on etiology and three aEEG indices would allow pediatricians to assess the risk for neurodevelopmental impairment and facilitate an early intervention in newborns developing seizures.

## Introduction

Seizures occur more frequently in the neonatal period than at any other time during the human lifespan. Population based studies suggest that the incidence of seizures in term neonates is 1 to 3 per 1000 live births [1]. Clinical recognition of neonatal seizures is usually difficult, because manifestations may be subtle and seizures are frequently not accompanied by any identifiable clinical symptoms [2]–[4]. Electroclinical dissociation is common in neonatal seizures i.e., the clinical seizures cease while electrographic seizures may persist after administration of antiepileptic drugs [3]. As a result, detection and identification of seizures by visual clinical observation alone is insufficient and unreliable in neonates [3]–[5]. Neonatal seizures should be evaluated using electroencephalogram (EEG) monitoring.

Multichannel video-EEG is currently considered as the “gold standard” for diagnosis of neonatal seizures. However, continuous video-EEG monitoring as well as its post-recording waveform interpretation is labor intensive so it is not practical in the majority of neonatal units around world [6]. As an alternative, amplitude-integrated EEG (aEEG) has been employed with the goal of early identifying and more aggressively treating neonatal seizures. The aEEG technique makes use of ongoing EEG amplitudes in single channel. In brief, the raw EEG signals from biparietal electrodes are amplified, filtered, and compressed over long periods of time to obtain a simplified EEG waveform that enables evaluation of long-term trends in electrocortical background activity [7]. Neonatal seizures are characterized as an abrupt and transient rise in the lower and upper margins of the aEEG tracing [2], [6].

There is a large body of literature on the use of aEEG for prediction of prognosis of neonates with encephalopathy [8]–[11], and asphyxia [12], [13]. A strong association between the early abnormal aEEG and impaired neurologic outcome has been established [14]–[18]. For instance, Hellström-Westas et al. [19] found that the aEEG pattern recorded within six postnatal hours successfully predicted the neurodevelopmental outcome in 47 asphyxia infants. Eken et al. [20] compared the prognostic value of five non-invasive techniques in infants with hypoxic-ischemic encephalopathy (HIE); the results suggested that aEEG method had the highest positive and negative predictive value for the subsequent outcome.

While the prognostic value of aEEG is well established in neonates with encephalopathy and asphyxia, so far as we know, neonatal seizure studies focusing on the direct correlation between early aEEG measurement and subsequent neurologic outcome are scare (one exceptional study examined the relation between aEEG and outcome in neonates with recurrent seizures [21]. On one hand, it has been widely recognized that both EEG features (e.g., interictal pattern [22]) and seizure etiology (e.g., global cerebral hypoxia-ischemia [23]) are critical in determining outcome and prognosis [24]–[26]. One the other hand, relevant aEEG studies in neonates have identified that the background pattern, cyclicity, and seizure activity of aEEG are good prognostic indices for subsequent outcomes [13], [16]–[18], [21], [27], [28]. Accordingly, this study investigated the relationship between the neurodevelopmental outcome at one year of age in neonates developing seizures and its four predictors obtained during the neonatal period (i.e., the etiology of neonatal seizures and three aEEG indices). Since the interpretation agreement among different aEEG raters affects the reliability of the proposed prognostic system, the interrater agreement was also evaluated in this study. We retrospectively examined the aEEG recordings of full-term neonates diagnosed with neonatal seizures based on multi-channel EEG findings. The prognostic value of aEEG features was systematically analyzed with the purpose to identify a reliable predictive system that facilitates the evaluation of neonate’s risks for neurodevelopmental impairment after neonatal seizures.

## Methods

### Patients

In total, 143 neonates (82 males) were retrospectively enrolled based on admission or discharge diagnoses of “neonatal seizures”. The inclusion criteria were: (1) the patients who were admitted to the neonatal ward of Peking University First Hospital in Beijing, between November 1, 2007 and March 31, 2012; (2) birth gestational age (GA)≥37 weeks and birth weight appropriate for GA; (3) clinically evident neonatal seizures at least once within the first 28 days of life; (4) at least one electrographic or electroclinical seizure presence on multi-channel EEG within the first 28 days of life; (5) neurologic follow-up to at least 12 months of age.

Diagnosis of clinical neonatal seizures was based on observations by nurses or other ward staffs and confirmed by a pediatric neurologist (X Hou). Diagnosis of electrographic seizures was performed independently by an experienced neurophysiologist who was at present of conventional multi-channel EEG recording.

The average postmenstrual ages (PMAs), calculated by adding the weeks of GA to the postnatal age, were 41.0±1.9 weeks and 41.1±2.0 weeks, respectively, when full-channel EEG and aEEG data were registered. Informed consent was signed by the parent or legal guardian of the infants to approve the use of patient information and EEG/aEEG data for scientific purposes. The research protocol was approved by the Ethics Committee of Peking University.

### Seizure Management

A consistent institutional protocol was used for neonatal seizure management based on published guidelines [22]. In brief, the first-line AED was phenobarbital with a loading dose of 20 mg/kg. Only clinically visible seizures were treated. We considered treatment with antiepileptic drugs to have been successful if the seizures stopped and did not recur clinically.

### Etiology

The primary etiology of the neonatal seizures was determined mainly based on case history, clinical manifestation, physical examination, laboratory tests, and imaging studies including magnetic resonance imaging, computed tomographic scan and cerebral ultrasound scan.

For the convenience of statistical analysis, etiologies were grouped into the following three levels. Level 0 of etiologies was associated with favorable outcome while Level 2 was associated with poor outcome [22], [24]–[26], [29], [30].

Level 0: transient metabolic abnormalities; mild HIE; IVH of degree I; benign idiopathic neonatal seizures; benign familial neonatal seizures.

Level 1: moderate HIE; IVH of degree II; cerebral infraction (small range); general sepsis; bacterial meningitis (without complication); subarachnoid hemorrhage.

Level 2: severe HIE; IVH of degree III and IV; cerebral infraction(wide range or multiple); brain malformation; bacterial meningitis (with complications); recurrent hypoglycemia; severe epilepsy syndrome; congenital metabolic syndrome (enzymatic disorder).

### aEEG Recording

In our neonatal unit, newborns with evidence of clinical seizures were examined using multi-channel EEG within 24 h of seizure observation. Further, newborns with EEG confirmation of seizures routinely underwent at least one follow-up aEEG examination within 48 h of EEG registration. Therefore, the aEEG data were available for all the neonates included in this study as part of the routine assessment. The sleep aEEG monitoring continued for at least 90 min and covered a complete cycle of quiet sleep (QS) and active sleep (AS). When the sleep state changes were not clearly distinguishable, the recording continued for at least 60 min. To minimize the influence of antiepileptic drugs or central sedative medication, no antiepileptic or other medications that potentially affect the central nervous system was administered for at least 2 h before aEEG registration.

EEG data were recorded using a bioelectric amplifier (Symtop Instrument Co, Beijing, China). The aEEG tracings were calculated according to the algorithm previously described by Zhang et al. [31], which has been verified by employing a commercially available aEEG monitor (Olympic CFM 6000, Natus, Seattle, WA). Three Ag-AgCl-disk electrodes were attached to the scalp for noninvasive registration by uing Ten20 conductive paste (Weaver and Company, Aurora, USA). Two detecting electrodes were fixed in P3 and P4 in the international 10–20 system, and a ground electrode was placed in Fz. The impedance was kept below 5 kΩ during the recording. Observed clinical seizures and handling of the infant were recorded by the nursing staff.

### Evaluation of aEEG Tracings

The aEEG data were appraised in respect to background pattern, appearance of cyclicity, and seizure activity, scoring from 0 (normal) to 2 (severely abnormal).

#### Background pattern

This aEEG feature describes the dominating type of aEEG tracings. The background pattern was classified according to the method previously described by Hellström-Westas et al. [7], [11], [19].

Score 0 (normal): continuous tracing with a maximum voltage of 10 to 50 µV and a minimum voltage between 5 to 10 µV.

Score 1 (mildly abnormal): discontinuous tracing with voltage predominantly >5 µV.

Score 2 (severely abnormal): burst-suppression pattern with periods of very low voltage without variability (<5 µV) intermixed with bursts of higher amplitude (>25 µV); continuous low voltage with continuous background and maximum voltage around or below 5 µV; flat trace with inactive background and very low voltage below 5 µV.

#### Cyclicity

The cyclicity of full-term aEEG tracing is characterized by smooth periodic changes in aEEG bandwidth. The broad and high bandwidth represents relatively discontinuous background activity during QS while the narrow and low bandwidth corresponds to the more continuous activity during AS. The aEEG cyclicity was classified with regard to the bandwidth variations of the tracings.

Score 0 (mature): clearly identifiable variations by more than 2 µV and with a cyclic duration of ≥20 min [15].

Score 1 (immature): imminent cyclicity displayed less clear variations in the lower margin between sleep stages as compared with the fully developed mature tracing.

Score 2 (none): absence of cyclicity.

#### Seizure activity

Seizures in aEEG were defined as a characteristic pattern with sudden increases of both the lower and upper margins (or sometimes only the lower margin) of the tracing. The seizure activity in aEEG was first verified by inspecting the simultaneously presented raw EEG and then was scored in three levels.

Score 0: none or single seizure.

Score 1: repetitive seizures with ≥3 discharges during a 30-min period.

Score 2: status epilepticus presenting as a “sawtooth pattern” or as continuous increases of the lower and upper margins.

All aEEG tracings were visually rated by two of the authors (H Ding and X Hou) blinded to the patient identity and clinical data. Interrater reliability was determined between these two readers. When there was discrepancy about aEEG scores between the two raters, a third rater (D Zhang) was involved and consensus was reached. All the three aEEG readers had full access to marked events and simultaneous raw EEGs.

### Assessment of Neurodevelopmental Outcome

The neurodevelopmental outcomes of infants were examined at one year of age by an experienced pediatrician unaware of the aEEG findings (C Zhou). Gesell Developmental Scale (GDS) for 0- to 6-year-old children was employed to assess infants’ cognition and behavioral development with a domestic adjustment by the Chinese Pediatric Association [32]. In particular, the GSD was designed to assess the development quotient (DQ) of infants associated with motor skills, adaptive capacity, language, and personal–social skills. The neurodevelopmental outcomes of patients were classified into three levels according to DQ scores and other abnormal neurologic signs [10], [27].

Level 0: normal neurodevelopment with DQ>75.

Level 1: mild to moderate cognitive impairment with DQ≥40 and ≤75, hearing impairment with no amplification, or persistent seizure disorder at one year of age.

Level 2: severe cognitive impairment with DQ<40, hearing impairment requiring hearing aids, blindness, or cerebral palsy.

### Statistical Analysis

Statistical analyses were performed using SPSS Statistics 17.0 (IBM, Somers, USA). Statistical significance was assumed for *p*<.05.

#### Univariate analysis

Etiologic and aEEG scores were qualitatively compared for infants experiencing favorable, moderate, and adverse outcomes by means of the Pearson Chi-Square (χ^2^) or Fisher’s exact test as appropriate (3×3 comparisons for categorical variables) [21], [33]. Odds ratio (OR) calculations were performed to evaluate the predictive value of the four prognostic indices for the adverse outcome (i.e., outcomes evaluated with Score 2) [10], [16], [34], [35].

#### Multivariate analysis

Since the neurological outcome was analyzed as an ordinal variable (0–1–2) in this study, ordinal logistic regression was performed to determine the effect of the four independent variables on neurological development at one year of age (dependent variable). To fulfill the Test of parallel lines, the link function of Probit was used.

Interrater agreement between the two aEEG readers was determined using kappa concordance test. Cohen’s kappa coefficient (κ) was calculated based on the aEEG rating scores of background pattern, cyclicity, and seizure activity, respectively.

## Results

### Etiologic Distribution

A total of 143 full-term neonates were enrolled in this study. Neonatal seizures were confirmed using multi-channel EEG in all these newborns. The etiology of the neonatal seizures was determined during neonatal period based on case history, clinical manifestation, laboratory tests, imaging studies, etc (Table 1). In this study, the most common etiologies of neonatal seizures were HIE (n = 44), intraventricular hemorrhage (IVH) (n = 18), and cerebral infraction (n = 12). According to previous findings on the relationship between seizure etiology and subsequent neurologic outcome [24], [25], [29], [30], etiologies were grouped into three levels.

**Table 1:** Etiologic distribution of neonatal seizures in this study.

Etiology	n (%)
**Level 0** (a potential favorable outcome)	**42 (29%)**
transient metabolic abnormalities	5 (3%)
mild HIE	21 (15%)
IVH of degree I	7 (5%)
benign idiopathic/familial neonatal seizures	9 (6%)
**Level 1** (a potential moderate outcome)	**42 (29%)**
moderate HIE	16 (11%)
IVH of degree II	6 (4%)
cerebral infraction (small range)	7 (5%)
general sepsis	5 (3%)
bacterial meningitis (without complication)	4 (3%)
subarachnoid hemorrhage	4 (3%)
**Level 2** (a potential adverse outcome)	**41 (29%)**
severe HIE	7 (5%)
IVH of degree III and IV	5 (3%)
cerebral infraction (wide range or multiple)	5 (3%)
brain malformation	6 (4%)
bacterial meningitis (with complications)	3 (2%)
recurrent hypoglycemia	4 (3%)
severe epilepsy syndrome	7 (5%)
congenital metabolic syndrome	4 (3%)
**etiology unknown**	**18 (12%)**

HIE: hypoxic-ischemic encephalopathy, IVH: intraventricular hemorrhage.

### Neurodevelopmental Outcome

The DQ score in patients with normal neurodevelopmental outcomes (Level 0) was 87.2±6.9 (mean±SD). Among them, 18 patients have marginal normal outcomes (i.e., DQ = 76 – 85). The DQ scores were 60.8±9.0 and 30.0±6.9 in patients with mild to moderate (Level 1) and severe cognitive impairments (Level 2). There were 16 infants developed cerebral palsy; 5 had serious hearing impairment; and 1 patient was blind at the age of 9 month.

### aEEG Pattern, Cyclicity, and Seizure Activity

The sleep aEEG examination was performed in the 143 newborns in the neonatal period. Although aEEG recordings were initiated at the periods without clinically recognized seizures (i.e., interictal periods), clinical seizures were observed in 19 patients (19/143) during aEEG monitoring. The current study focused on three aEEG features that potentially provide prognostic value for neurodevelopmental outcomes of neonatal seizures. The background pattern, cyclicity, and seizure activity of aEEG tracings were scored from 0 (normal) to 2 (severely abnormal) (refer to Table 2). It has been reported that the neonates without clear etiology usually had good neurodevelopmental outcomes [26], [30], [34]. For the sake of statistical convenience, the unknown etiology (n = 18, Table 1) was grouped into the seizure etiology Level 0 in the statistical analyses in this study (i.e., etiologies associated with favorable outcome).

**Table 2:** aEEG and etiology scores obtained at neonatal period associated with outcome findings at the age of one year.

	Neurological outcome			?^2^(4)	*p* b
Predictor	0	1	2		
	(n = 49)	(n = 43)	(n = 51)		
Etiology				79.3	.000
0	38 (78)	13 (30)	9 (18)		
1	10 (20)	24 (56)	8 (16)		
2	1 (2)	6 (14)	34 (67)		
aEEG background				116.9	.000
0	41 (84)	12 (28)	2 (4)		
1	8 (16)	27 (63)	11 (22)		
2	0 (0)	4 (9)	38 (74)		
aEEG cyclicity				87.2	.000
0	31 (63)	12 (28)	2 (4)		
1	18 (37)	25 (58)	11 (22)		
2	0 (0)	6 (14)	38 (74)		
aEEG seizure				34.4^a^	.000
0	45 (92)	28 (65)	22 (43)		
1	4 (8)	15 (35)	20 (39)		
2	0 (0)	0 (0)	9 (18)		

a Fisher’s exact test;

b two-sided *p* value. Data are shown as n (%). Outcome scores, 0: normal, 1: mildly or moderately abnormal, 2: severely abnormal. Etiology scores, 0: associated with a potential favorable outcome, 1: associated with a potential moderate outcome, 2: associated with a potential adverse outcome. aEEG background pattern scores, 0: normal, 1: mildly abnormal, 2: severely abnormal. aEEG cyclicity scores, 0: mature, 1: immature, 2: none. aEEG seizure scores, 0: none or single seizure, 1: repetitive seizures, 2: status epilepticus.

**Figure 1:**
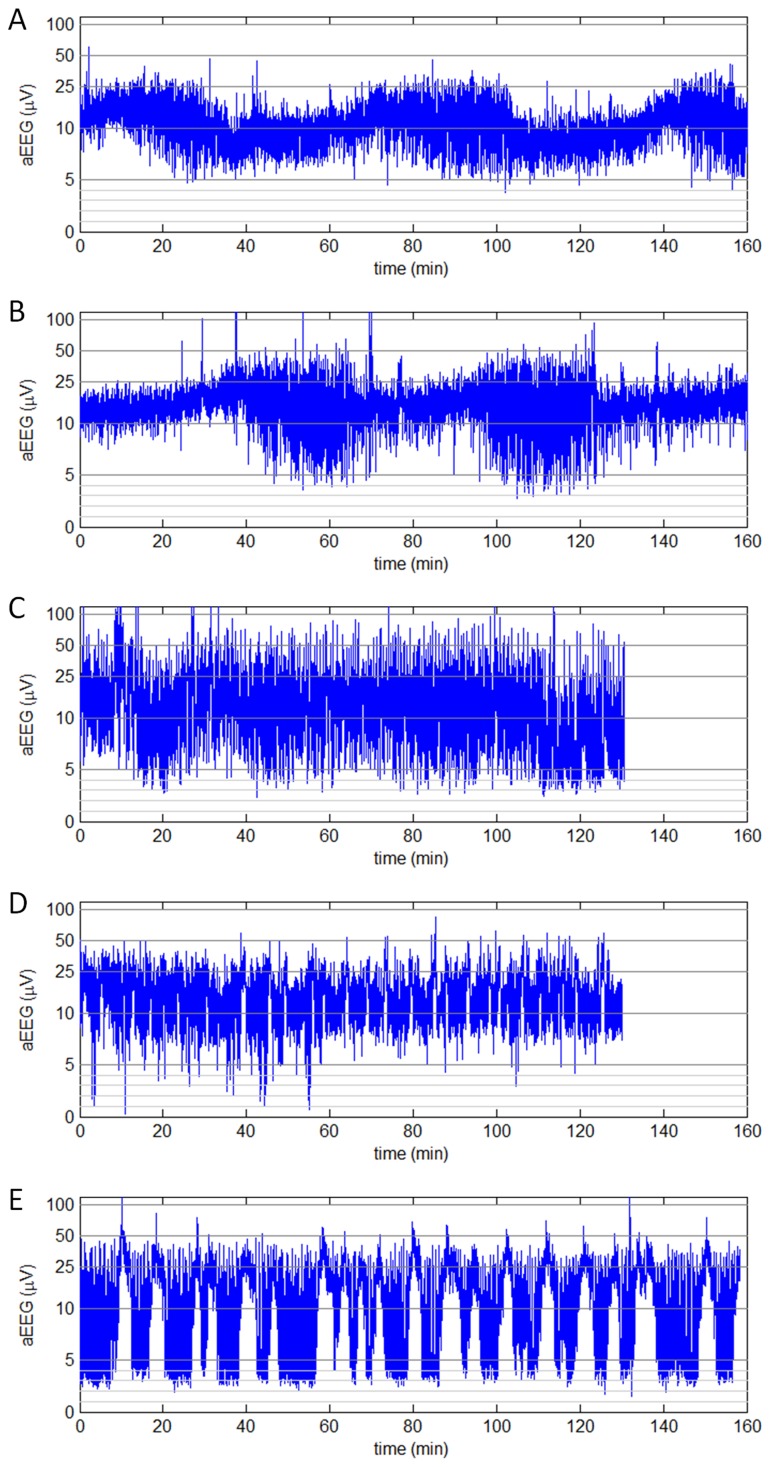
Characteristic appearance of aEEG tracings with different scores of background pattern, cyclicity, and seizure activity. *A*, background pattern = 0, cyclicity = 0, seizure activity = 0; postmenstrual age (PMA) = 39 weeks+6 days. *B*, background pattern = 0, cyclicity = 0, seizure activity = 0; PMA = 39w+4. *C*, background pattern = 1, cyclicity = 0, seizure activity = 0; PMA = 37w+3. *D*, background pattern = 0, cyclicity = 1, seizure activity = 1; PMA = 43w+2. *E*, background pattern = 2, cyclicity = 2, seizure activity = 2; PMA = 39w+6.

Among the 143 aEEG recordings, 55 were classified as normal background pattern (Score 0) with appropriate voltages in lower and upper margins for full-term neonates (Figure 1A, B, D) [7]. Mildly abnormal aEEG pattern with discontinuous waves (Score 1) was found in 46 patients (Figure 1C). The other 42 aEEG recordings were classified as severely abnormal background (Score 2), including burst-suppression (Figure 1E), low voltage, and inactive aEEG patterns.

Mature aEEG cyclicity in full-term neonates was defined as with identifiable waveform variations and with a cyclic duration of ≥20 min (Score 0, Figure 1A, B, C) [15]. The mature cyclicity was found in 45 out of 143 aEEG tracings. Immature aEEG cyclicity (Score 1) displayed less clear variations as compared with the fully developed mature aEEG and was observed in 54 neonatal aEEG recordings (Figure 1D). No voltage difference appeared between different sleep stages in 44 aEEGs, which were classified as no cyclicity (Score 2, Figure 1E).

Among the 143 aEEG recordings, 95 were with no or single seizure (Score 0, Figure 1A, B, C), since aEEG examination was initiated during interictal period. Repetitive seizures (Score 1) were observed in 39 aEEG tracings (Figure 1D) and status epilepticus activity (i.e., “sawtooth” aEEG; Score 2) was found in 9 aEEG tracings (Figure 1E).

Finally, multicollinearity diagnostics were performed among the three aEEG indices. The result showed that condition indices were no more than 5.0 (from 2.0 to 5.0). Spearman correlation coefficients among aEEG indices were as follows: *r* = 0.66 between aEEG pattern and cyclicity; *r = *0.36 between aEEG pattern and seizure activity; and *r* = 0.32 between aEEG cyclicity and seizure activity (*p*s<0.01).

### Relations between Neonatal aEEG and Neurodevelopmental Outcome at one Year

The postnatal ages of detection of clinical seizures in the three outcome groups did not show significant difference (*F*(2,140)<1, *p* = 0.493; outcome 0 = 5.5±6.3 days; outcome 1 = 7.8±11.0 days; outcome 2 = 7.2±11.0 days).

Univariate comparisons were performed between the neurological outcome scores at the age of one year and the etiologic and aEEG scores obtained within the first 4 weeks of life (Table 2 and 3). Results of the Pearson Chi-Square revealed that aEEG background pattern was highly associated with neurologic outcomes (χ^2^(4) = 116.9, *p*<.001), followed by aEEG cyclicity (χ^2^(4) = 87.2, *p*<.001) and seizure etiology (χ^2^(4) = 79.3, *p*<.001). Odds ratio (OR) calculations suggested that severely abnormal aEEG background pattern (aEEG background Score 2) had the highest predictive value for severely abnormal outcome (i.e., outcomes evaluated with Score 2) (OR = 251.8, *p*<.001); absence of aEEG cyclicity (aEEG cyclicity Score 2) had the next highest predictive value for severely abnormal outcome (OR = 136.2, *p*<.001).

**Table 3:** The odds ratio (OR) of aEEG and etiology scores associated with outcome findings at the age of one year.

Predictor	OR result (95% CI)		
	Outcome 0+1 vs. 2^a^	Outcome 0 vs. 1+2^b^	Outcome 0 vs. 1
Etiology			
0	1.0	1.0	1.0
1	1.3 (0.5–3.8)	5.5 (2.3–13.4)	7.0 (2.7–18.5)
2	27.5 (9.4–81.0)	69.1 (8.9–538)	17.5 (1.9–159.7)
aEEG background			
0	1.0	1.0	1.0
1	8.3 (1.7–39.9)	13.9 (5.2–36.8)	11.5 (4.2–31.9)
2	251.8 (43.8–1445.5)	n/a	n/a
aEEG cyclicity			
0	1.0	1.0	1.0
1	5.5 (1.2–26.3)	4.4 (1.9–10.3)	3.6 (1.4–8.8)
2	136.2 (25.9–715.2)	n/a	n/a
aEEG seizure			
0	1.0	1.0	1.0
1	3.5 (1.6–7.7)	7.9 (2.6–23.9)	6.0 (1.8–20.0)
2	n/a	n/a	n/a

a For OR calculation, neonates with the outcome scores of 0 and 1 were combined as a single outcome group; the etiology and aEEG features were compared between Score 0 and Score 1, and between Score 0 and Score 2, respectively.

b Neonates with the outcome scores of 1 and 2 were combined as a single outcome group. CI, confidence interval. n/a, not applicable.

For convenient comparison with results of relevant studies, the prognostic value of the aEEG and etiology indices was further investigated by calculating the conventional statistics of sensitivity, specificity, and positive and negative predictive value (Table 4 and 5).

**Table 4:** The predictive value of aEEG features and etiology for severely abnormal prognosis in neonates with seizures.

Predictor	Sensitivity(%)	Specificity(%)	PPV(%)	NPV(%)
Cut off = 2				
Etiology	66.7	92.4	82.9	83.3
aEEG background	74.5	95.6	90.5	87.1
aEEG cyclicity	74.5	93.5	86.4	86.9
aEEG seizures	17.6	100	100	68.7
Cut off = 1				
Etiology	82.4	55.4	50.6	85.0
aEEG background	96.1	57.6	55.7	96.4
aEEG cyclicity	96.1	46.7	50.0	95.6
aEEG seizures	56.9	79.4	60.4	76.8

PPV, positive predictive value. NPV, negative predictive value. Normal and mildly/moderately abnormal outcomes were put together as one outcome group. Sensitivity = true positive/(true positive+false negative); specificity = true negative/(true negative+false positive); positive predictive value = true positive/(true positive+false positive); negative predictive value = true negative/(true negative+false negative).

**Table 5:** The predictive value of aEEG features and etiology for abnormal prognosis in neonates with seizures.

Predictor	Sensitivity(%)	Specificity(%)	PPV(%)	NPV(%)
Cut off = 2				
Etiology	42.6	98.0	97.6	47.1
aEEG background	44.7	100	100	48.5
aEEG cyclicity	46.8	100	100	49.5
aEEG seizures	9.6	100	100	36.6
Cut off = 1				
Etiology	76.6	77.6	86.8	63.3
aEEG background	85.1	83.7	90.9	74.6
aEEG cyclicity	85.1	63.3	81.6	68.9
aEEG seizures	46.8	91.8	91.7	47.4

Mildly/moderately abnormal and severely abnormal outcomes were put together and only “normal *vs*. abnormal” was analyzed.

Finally, multiple linear regression was performed to determine the effect of the four independent variables on neurological development at one year of age. Regression analysis showed that the predictive model provided a good fit to the data with a highly significant *F* value (*F*(4,138) = 88.6, *p*<.001), and that the four predictors explained 71.2% of the variation in neurodevelopmental outcomes (adjusted *R* ^2^ = 0.712). Results in Table 6 indicated that aEEG background pattern was the most significant independent variable to affect the regression model and the most strongly related to the neurological outcome at one year (standardized β = 0.44).

**Table 6:** Multiple linear regression model for neurodevelopmental outcome (measured as continuous variable of DQ score) by using aEEG features and etiology as predictors.

Predictor	β^a^	95% CI	*t*	*p*
etiology	0.22	0.11–0.33	3.9	.000
aEEG background	0.44	0.32–0.59	6.5	.000
aEEG cyclicity	0.24	0.11–0.39	3.6	.000
aEEG seizures	0.14	0.06–0.33	2.9	.005

a standardized β coefficient.

### Interrater Agreement of aEEG Rating

Interrater agreement of the aEEG rating for background pattern, cyclicity, and seizure activity was quantified using kappa coefficient (κ). Overall good agreement was found between the two aEEG readers (κ = 0.74, *p*<.001). The agreement for background pattern, cyclicity, and seizure activity was 0.77, 0.62, and 0.76, respectively (*p*s<.001).

## Discussion

The current study examined the aEEG data registered in a group of full-term newborns who presented EEG-confirmed neonatal seizures. Our data revealed that the neonatal aEEG features of background pattern, cyclicity, and seizure activity were significantly associated with the neurodevelopmental outcomes at the age of one year. This result was largely consistent with previous EEG studies, which reported that EEG features, especially background EEG pattern, correlate well with neurological outcome in neonates with seizures [5], [22], [23], [34], [36].

Many researchers found that interictal aEEG activity was associated with the subsequent neurologic outcome of encephalopathic neonates accompanied by seizures [8], [28]. However, very few studies have focused on neonates with seizures and systematically investigated the predictive value of aEEG features for neurodevelopmental outcomes. The most relevant study we’ve found so far was undertaken by van der Heide et al. [21], which showed that the aEEG background pattern was significantly associated with subsequent neurologic outcome in neonates treated with two or more antiepileptic drugs. It is suggested that pattern recognition forms the base of aEEG interpretation [7]. The present data showed that aEEG background pattern was the most informative predictor for the neurological outcome at one year, with a standardized β coefficient of 0.44 in a multiple regression model. Abnormal aEEG background pattern predicted adverse outcome with a sensitivity of 85%, a specificity of 84%, positive predictive value of 91%, and negative predictive value of 75%. These results were not only comparable with previous prognostic aEEG studies on neonatal encephalopathy and asphyxia (Table 7), but also provided a direct relation between early aEEG background pattern and subsequent neurologic outcomes in seizure-survived infants.

**Table 7:** Full-term neonatal studies on the predictive value of early abnormal aEEG background pattern for subsequent abnormal outcome (listed in time order).

Study	Focused neonates	n	Sensitivity(%)	Specificity(%)	PPV(%)	NPV(%)
Eken et al.	HIE	31	94	79	84	92
Hellström-Westas et al.	asphyxia	47	95	89	86	96
al Naqeeb et al.	encephalopathy	56	93	70	77	90
Toet et al.	asphyxia	68	91	86	86	91
Shalak et al.	high-risk	50	79	89	73	90
ter Horst	asphyxia	30	100	63	61	100
van Rooij et al.	asphyxia	160	93	85	88	91
Lavery et al.	encephalopathy	20	44	100	100	75
Thoresen et al.	asphyxia	31	80	73	84	67
Shankaran et al.	HIE	108	89	33	56	75
Massaro et al.	encephalopathy	69	83	93	87	91
van der Heide et al.	seizure	76	67	93	94	65
This study (cut off = 2)^a^	seizure	143	74	96	90	87
This study (cut off = 1)^b^	seizure	143	85	84	91	75

a severely abnormal aEEG background pattern (aEEG background pattern Score 2) predicted for severely abnormal outcomes (outcome Score 2).

b abnormal aEEG background pattern (aEEG background pattern Scores 1 and 2) predicted for abnormal outcomes (outcome Scores 1 and 2).

Our data also indicated that aEEG cyclicity was a useful independent predictor of the prognosis of infants with a history of neonatal seizures (standardized β = 0.24 in regression model). The presence of cyclicity on aEEG tracing is a sign of brain integrity in neonates. In contrast, absent cyclicity is a common characteristic finding indicating suppressed brain function and a high risk of cerebral injury [15]. Many studies in HIE and asphyxiated neonates have proved that a delayed onset of aEEG cyclicity was associated with poor neurodevelopmental outcome [12], [27]. Consistently, we found in this study that absence of aEEG cyclicity had a high predictive value for severely abnormal outcome in seizure-confirmed neonates (OR = 136.2).

Compared with aEEG background pattern and cyclicity, the seizure activity in aEEG tracing contributed less to prognosis estimation (standardized β = 0.14). It is understandable, because all the aEEG recordings were initiated during interictal period in this study; thus aEEG seizure activity was not observed in most of the neonates (95 out of 143). We preferred to investigate aEEG features in interictal period because it is believed that conventional EEG is always the gold standard for seizure detection and prognostic estimation [2], and that aEEG monitoring would have a promising application in neonates with clinical suspicion for seizures but when EEG with full neonatal array seems not necessary.

The seizure etiology was investigated in this study since it has been proved that association between seizure etiology and neurological prognosis is strong [22], [23], [25], [34]. We found that severe HIE, IVH, cerebral infraction, and structural brain malformations were responsible for the large majority of infants with the worst prognosis (OR = 27.5), which was consistent with most of the previous findings [23], [25]. However, there were some researchers suggested that seizure etiology was not associated with subsequent neurologic outcome and the risk for post-neonatal epilepsy [21], [36]. We think the discrepancy in the predictive value of seizure etiology might be due to the differences in inclusion criteria, outcome evaluation, and illness severity of the study populations [16].

Finally, it should be pointed out that the aEEG scoring system proposed in this study can be interpreted differently according to clinical purposes. The data in Table 4 and 5 indicated that on the one hand, the sensitivity was higher when considering the aEEG scores of 1 and 2 as abnormal results. On the other hand, the specificity was higher when considering the aEEG score of 2 as abnormal results. We prefer the first partition of aEEG score (0 vs. 1+2) because it provides a satisfactory sensitivity and alerts both clinical staffs and parents to closely examine the risk infants from as early as neonatal periods.

Our study had limitations. First, all the data were retrospectively investigated, which may induce inherent bias. Second, one of the inclusion criteria was clinical evidence of neonatal seizures. However, the reliance on clinical notes may cause an underestimation of clinical seizures if the nurses did not pay enough attention to suspicious patients. Third, imaging data was not included in this study. Fourth, no neonate with HIE was treated with hypothermia in this study. Although Massaro et al. [16] examined the aEEG data in newborns treated with hypothermia and concluded that aEEG background abnormality and absent cyclicity was associated with adverse outcome, more recent studies showed that aEEG has less predictive value in neonates with hypothermia treatment [10], [37]. This issue should be further examined and clarified in our further work.

In summary, to identify reliable prognostic indicators for neonates with seizures, we examined the relation between early aEEG recordings and neurodevelopmental outcomes in a relatively large population-based study. It is found that the aEEG features of background pattern, cyclicity, and seizure activity, as well as the seizure etiology were significantly associated with outcome. All these four indices were independent predictors in multiple regression model. In addition, very good interrater agreement was observed for aEEG assessment, suggesting that the aEEG tracing could be interpreted accurately by clinical staffs after a modest period of training [8]. This clinically applicable scoring system based on etiology and three aEEG indices would allow pediatricians to assess the risk for neurodevelopmental impairment and facilitate an early intervention in newborns developing seizures.
